# Interleukin 4 Moderately Affects Competence of Pluripotent Stem Cells for Myogenic Conversion

**DOI:** 10.3390/ijms20163932

**Published:** 2019-08-13

**Authors:** Barbara Świerczek-Lasek, Jacek Neska, Agata Kominek, Łukasz Tolak, Tomasz Czajkowski, Katarzyna Jańczyk-Ilach, Władysława Stremińska, Katarzyna Piwocka, Maria A. Ciemerych, Karolina Archacka

**Affiliations:** 1 Department of Cytology, Faculty of Biology, University of Warsaw, Miecznikowa 1 Str, 02-096 Warsaw, Poland; 2 Laboratory of Cytometry, Nencki Institute of Experimental Biology, Polish Academy of Sciences, Pasteur 3 Str, 02-093 Warsaw, Poland

**Keywords:** embryonic stem cells, pluripotency, differentiation, myogenesis, fusion, interleukin 4, hybrid myotubes

## Abstract

Pluripotent stem cells convert into skeletal muscle tissue during teratoma formation or chimeric animal development. Thus, they are characterized by naive myogenic potential. Numerous attempts have been made to develop protocols enabling efficient and safe conversion of pluripotent stem cells into functional myogenic cells in vitro. Despite significant progress in the field, generation of myogenic cells from pluripotent stem cells is still challenging—i.e., currently available methods require genetic modifications, animal-derived reagents, or are long lasting—and, therefore, should be further improved. In the current study, we investigated the influence of interleukin 4, a factor regulating inter alia migration and fusion of myogenic cells and necessary for proper skeletal muscle development and maintenance, on pluripotent stem cells. We assessed the impact of interleukin 4 on proliferation, selected gene expression, and ability to fuse in case of both undifferentiated and differentiating mouse embryonic stem cells. Our results revealed that interleukin 4 slightly improves fusion of pluripotent stem cells with myoblasts leading to the formation of hybrid myotubes. Moreover, it increases the level of early myogenic genes such as *Mesogenin1*, *Pax3*, and *Pax7* in differentiating embryonic stem cells. Thus, interleukin 4 moderately enhances competence of mouse pluripotent stem cells for myogenic conversion.

## 1. Introduction

Pluripotent stem cells (PSCs), such as embryonic stem cells (ESCs) or induced pluripotent stem cells (iPSCs), are characterized by unique properties, i.e., the ability of extensive proliferation, self-renewal, as well as multidirectional differentiation enabling generation of all mammalian cell types. For this reason, PSCs are considered as a potential source of cells that could be transplanted into defective tissues or organs to replace lost, damaged or malfunctioning cells. The first clinical trials using PSC derivatives have already been conducted and reported [1]. Among conditions that could be treated with PSC derivatives are skeletal muscle dysfunctions which may result inter alia from severe physical trauma or progression of skeletal muscle diseases, such as muscular dystrophies or spinal muscle atrophy. To reach this aim PSCs must be converted into functional myogenic cells with high efficiency and, preferentially, under defined culture conditions. 

PSCs present naive myogenic potential as they form skeletal muscle tissue in vivo, during teratoma formation or chimeric animal development [2,3]. However, despite numerous attempts, efficient in vitro conversion of PSCs into functional myogenic cells had been challenging until factors involved in embryonic myogenesis were used to promote PSC myogenic differentiation [4]. Activation of canonical Wnt, followed by fibroblast growth factor 2 (FGF-2) treatment led to the derivation of myogenic cells with high efficiency, i.e., nearly 90% of cells derived from mouse or human ESCs synthesized myogenic markers—such as Pax3, the marker of skeletal muscle precursor cells formed during embryonic myogenesis, or myosin heavy chains (MHC)—characteristic of already committed and differentiated myogenic cells [5]. However, the generation of such cells required 50-day long culture [5]. Moreover, their myogenic potential has not been verified in vivo. In the study by Chal and co-workers, the activation of canonical Wnt signaling with simultaneous BMP4 inhibition enabled the conversion of PSCs into multinucleated striated myofibers, i.e., basic structural and functional skeletal muscle units, which were accompanied by cells expressing Pax7, i.e., marker characteristic of satellite cells, skeletal muscle stem cells responsible for muscle growth and regeneration. Transplantation of Pax7-positive cells into *mdx* mice, the animal model of Duchenne muscular dystrophy (DMD), led to the appearance of dystrophin, a protein which is not present in DMD muscle, causing myofiber membrane fragility and muscle injuries. Moreover, transplanted cells settled in the satellite cell niche, which is crucial for effective and long lasting cell therapy for dysfunctional skeletal muscles [6]. Altogether, several protocols have already demonstrated that myogenic differentiation of PSCs can be successfully supported by myogenesis regulators such as FGF-2, Wnts, and other factors [7]. However, indicated methods are usually time-consuming and, therefore, should be further improved to be even more efficient, faster, safer, and conducted under defined culture conditions. 

In the current study, we focused on the influence of interleukin 4 (IL-4) on the fate of PSCs, mainly their competence for myogenic conversion. IL-4 plays an important role in myogenesis as it is engaged in recruiting myoblasts—i.e., mononuclear committed myogenic cells—into nascent multinuclear myotubes, leading to the increase in nuclei number in myotubes, their growth, and eventually myofiber formation. This process is critical for proper development and maintenance of functional skeletal muscles because, as mentioned above, multinucleated myofibers are their basic units. In skeletal muscle, IL-4 acts through the type II receptor, comprised of IL-4Rα and IL-13Rα1 [8]. Lack of either IL-4 or IL-4Rα receptor subunit in mouse skeletal muscle results in the decreased number of nuclei in myofibers, and—as a consequence—smaller and thinner muscles of such knockout mice [8]. Similar effect was observed during postnatal development of mice lacking serum response factor (SRF), a transcription factor regulating expression of different genes crucial for proper muscle function such as dystrophin, muscle creatinine kinase, or insulin-like growth factor 1 (IGF-1). Disruption of the *Srf* gene results in strong downregulation of IL-4 expression, impairing myoblast recruitment to myotubes and myofibers, delayed postnatal muscle growth, and decreased muscle mass [9]. The role of IL-4 is not limited to the skeletal muscle cells. This cytokine is also important for immune cells, as it modulates inter alia proliferation of B and T cells [9,10,11,12]. It was also shown that IL-4 promotes fusion of bone marrow derived mesenchymal stem cells (BM-MSCs) with myoblasts during their co-culture [13]. However, the mechanism by which IL-4 influences different cell types is only partially understood. It was reported that IL-4 induces expression of vascular cell adhesion molecule 1 (VCAM1) in eosinophils and basophils [14] and increases the expression of integrins and other cell membrane proteins in B cells [15] and macrophages [16]. Elevated expression of β1 and β3 integrins was also detected in myogenic cells treated with IL-4 which, as a result, were characterized by enhanced ability to migrate both in vitro and in vivo during skeletal muscle regeneration [17]. All these reports suggest that IL-4-mediated modulation of cells’ ability to adhere, migrate and fuse may be linked with the changes in the expression of cell membrane proteins, induced by this cytokine. 

Our previous study showed that undifferentiated mouse ESCs may fuse with myoblasts during their co-culture. As a result of it, ESC-derived nuclei found in hybrid myotubes formed by both ESCs and myoblasts are characterized by the presence of myogenic regulatory factors (MRFs), i.e., markers characteristic of committed myogenic cells [18]. However, this process occurs with a very low efficiency: hybrid myotubes make up to no more than 1% of all myotubes found in the co-culture of ESCs and myoblasts. In the same study, we also showed that undifferentiated ESCs lack proteins important for skeletal muscle myoblast fusion such as VCAM1 and M-cadherin, which may limit their competence for fusion [18]. On the basis of previously described data on IL-4’s role in myogenic and other cells, we investigated the influence of this cytokine on PSCs to verify whether IL-4 may enhance ESC ability to fuse with myoblasts as well as other features of these cells, e.g., proliferation rate or their gene expression profile. We analyzed both undifferentiated ESCs and cells cultured in embryoid bodies (EBs), i.e., three dimensional aggregates formed by PSCs differentiating in suspension culture, in medium lacking self-renewal promoting factors, such as leukemia inhibitory factor (LIF). 

## 2. Results

### 2.1. Expression of IL-4 and Its Receptors in Undifferentiated ESCs

**Figure 1:**
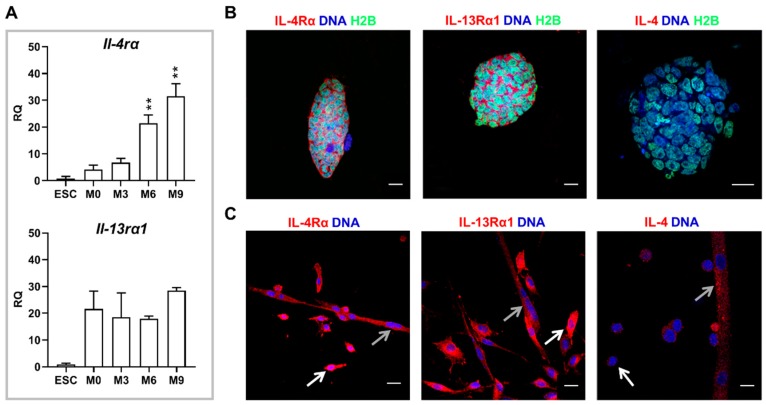
Analysis of expression of IL-4 and IL-4 type II receptor subunits in undifferentiated mouse ESCs and mouse primary myoblasts. (**A**) Expression of *Il-4rα* and *Il-13rα1* in undifferentiated ESCs and primary myoblasts analyzed at four stages of the culture: M0 (freshly isolated satellite cells), M3 (day 3 of the culture, predominately proliferating myoblasts), M6 (day 6 of the culture, predominately fusing myoblasts), and M9 (day 9 of the culture, predominately myotubes). *β-actin* was used as a reference gene. RQ = 1 for the level of gene expression detected in 13.5-day-old mouse embryo. Data are presented as the means of three independent experiments with standard deviations; ** *p* < 0.01. (**B**) Representative images of immunocytochemistry analysis of IL-4Rα, IL-13Rα1, and IL-4 in undifferentiated ESCs. Scale bar: 20 μm. (**C**) Representative images of immunocytochemistry analysis of IL-4Rα, IL-13Rα1, and IL-4 in mouse primary myoblasts at day 6 (white arrows indicate myoblasts, grey arrows indicate myotubes). Scale bar: 20 μm.

In the initial set of experiments, we checked whether undifferentiated ESCs are able to respond to IL-4, i.e., whether they synthesize receptors for IL-4. IL-4 may act through two types of receptors: type I, comprised by IL-4Rα and γC subunits, and present mostly in hematopoietic cells; and type II, comprised by IL-4Rα and IL-13Rα1 subunits, and found in other cell types, including myoblasts [19]. We assessed the expression of genes encoding IL-4Rα, IL-13Rα1, and γC in undifferentiated ESCs and in freshly isolated satellite cells (day 0; M0), satellite cell derived myoblasts at proliferation (day 3; M3), fusion (day 6; M6), and myotube (day 9 of the culture; M9) stages. We detected transcript encoding γC subunit only in bone marrow derived cells, which served as a positive control, but neither in undifferentiated ESCs nor myoblasts (data not shown). qPCR analysis revealed that both subunits of IL-4 type II receptor were expressed in undifferentiated ESCs, as well as in primary myoblasts at all stages of their differentiation (Figure 1A). The level of *Il-4rα* expression was significantly elevated in fusing myoblasts and myotubes in comparison to freshly isolated satellite cells and proliferating myoblasts, while no significant differences were observed in *Il-13rα1* expression level (Figure 1A). qPCR results were confirmed by immunostaining analyses showing that IL-4 type II receptor subunits are present in the membrane of both undifferentiated ESCs and primary myoblasts (Figure 1B,C). Distribution of both subunits in undifferentiated ESC colonies was heterogenous, i.e., in each colony there were cells characterized by the presence or absence of analyzed proteins (Figure 1B). Both receptor subunits were easily detectable in myoblasts (Figure 1C). In addition, we also checked whether undifferentiated ESCs and primary myoblasts synthesized endogenous IL-4. Immunocytochemistry analysis revealed that IL-4 was present only in multinucleated myotubes while it was not detected in neither proliferating nor fusing myoblasts as well as in undifferentiated ESCs (Figure 1B,C). Unfortunately, neither designed primers nor Taqman assays for *Il-4* yielded a specific PCR product. Therefore, IL-4 analysis was performed only at the protein level (Figure 1B,C). 

On the basis of the results described above, we concluded that at least some of undifferentiated ESCs are able to bind and respond to IL-4 but that these cells do not synthesize IL-4. 

### 2.2. Impact of IL-4 Treatment on Fusion of ESCs and Myoblasts

In the next set of experiments, we focused on the pivotal aim of our study, i.e., assessment of IL-4 influence on fusion of undifferentiated ESCs with myoblasts. To this end, we seeded undifferentiated ESCs on a layer of differentiating myoblasts and co-cultured both type of cells in medium containing 5 ng/mL of exogenous IL-4 or in control medium (i.e., without exogenous IL-4) for 3, 6, and 9 days (experimental scheme in Supplementary Figure S1A). In all co-culture experiments we used mouse C2C12 myoblasts, which are widely used as a model of myoblast differentiation and fusion. qPCR and immunocytochemistry analyses confirmed that C2C12 myoblasts expressed both subunits of IL-4 type II receptor, i.e., were capable of responding to this cytokine (data not shown). The co-culture of proliferating C2C12 myoblasts seeded on the layer of differentiating myoblasts served as a positive control, while the co-culture of differentiating myoblasts with mouse embryonic fibroblasts (MEFs), which do not present naive ability to fuse, served as a negative control. Importantly, we found that MEFs were characterized by negligible levels of mRNAs encoding IL-4 receptor subunits, thus were unlikely to respond to this cytokine (data not shown). The co-cultures of differentiating myoblasts with either MEFs or proliferating myoblasts were conducted under the same protocol as the co-cultures of ESCs and C2C12 myoblasts, i.e., cells were cultured in control medium or in the medium containing exogenous IL-4 and analyzed after 3, 6, and 9 days. 

**Figure 2:**
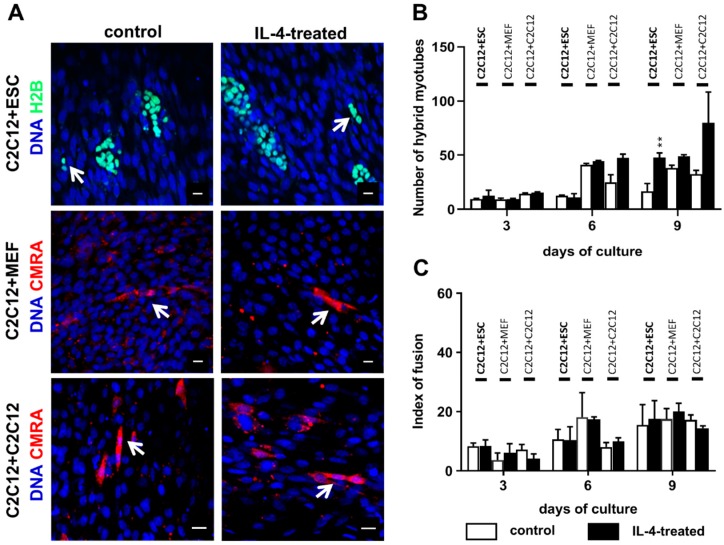
Analysis of IL-4 influence on fusion in the ESC-myoblast co-culture. (**A**) Representative images of hybrid myotubes in the co-cultures of differentiating C2C12 myoblasts with either ESCs (C2C12 + ESC), MEFs (C2C12 + MEF) or proliferating C2C12 myoblasts (C2C12 + C2C12) conducted with (IL-4-treated) or without (control) exogenous IL-4. White arrows indicate hybrid myotubes formed in each co-culture variant. Scale bar: 20 μm. (**B**) Number of hybrid myotubes formed in IL-4-treated and control co-cultures of C2C12 myoblasts with either ESCs, MEFs, or C2C12 myoblasts. Number of hybrid myotubes was calculated at day 3, 6, and 9 of the co-culture in three independent experiments and showed as the mean with standard deviations. ** *p* < 0.01 (**C**) Index of fusion in IL-4-treated and control co-cultures of C2C12 myoblasts with either ESCs, MEFs, or C2C12 myoblasts. Index of fusion was calculated at day 3, 6, and 9 of the co-culture in three independent experiments and showed as the mean with standard deviations.

To verify whether IL-4 improves fusion of undifferentiated ESCs with C2C12 myoblasts, we compared the number of hybrid myotubes formed by both cell types in the presence or absence of exogenous IL-4. Identification of hybrid myotubes was possible as we used ESCs expressing H2B-GFP fusion protein. Thus, hybrid myotubes contained ESC-derived nuclei marked by GFP fluorescence. Additionally, we performed the immunolocalization of skeletal muscle specific marker to visualize all myotubes in the co-culture (Supplementary Figure S2A). MEFs and proliferating C2C12 myoblasts used in control variants of the co-culture were first labeled with CMRA, a fluorescent cytoplasmic dye, and then seeded on the layer of differentiating myoblasts. Hybrid myotubes were found in all analyzed variants of the co-cultures (Figure 2A). The number of hybrid myotubes increased during the culture and was the highest after 9 days in all types of the co-culture (Figure 2B). It is worth noting that while the number of hybrid myotubes increased gradually in both types of control co-cultures, it did not change between day 3 and 6 of the ESC-myoblast co-culture, but then increased at day 9 (Figure 2B). We found that IL-4 treatment resulted in significantly higher number of hybrid myotubes only in the co-culture of C2C12 myoblasts with ESCs analyzed after 9 days of the culture (Figure 2B). It should be noted however, that hybrid myotubes were rare in all types of the co-culture and their contribution to total myotube number did not exceed 3% (Supplementary Figure S2B). Analysis of the fusion index revealed that it increased in all co-cultures, regardless of the presence or absence of exogenous IL-4 (Figure 2C). These results indicate that IL-4 does not influence the overall number of myotubes formed in any type of the co-culture, but selectively enhances the competence of ESCs to fuse with myoblasts, however to a moderate degree. To verify whether the observed effect is indeed caused by IL-4 treatment, we co-cultured ESCs and myoblasts with IL-4 along with antibodies targeting either IL-4 or IL-4Rα and found that under such conditions hybrid myotubes were virtually absent (Supplementary Figure S2A).

In addition, we also checked whether IL-4 impacted the proliferation of analyzed cells and found that the numbers of cells co-cultured in the presence or absence of exogenous IL-4 were similar (Supplementary Figure S3). IL-4 also did not impact the proliferation of cells cultured without differentiating C2C12 myoblasts (only ESCs, only MEFs, only proliferating C2C12 myoblasts; Supplementary Figure S3). Overall, these results show that IL-4 treatment does not impact proliferation of neither undifferentiated ESCs, MEFs, nor C2C12 myoblasts, either cultured separately or in co-culture.

### 2.3. Influence of IL-4 on Expression of Selected Cell Membrane Proteins in ESCs

Since previous results suggested that IL-4 treatment affects the competence of ESCs to fuse with myoblasts, we concluded that IL-4 may modulate expression of cell membrane proteins in ESCs. Thus, we looked at the expression of several selected factors engaged in cell adhesion and fusion in both control and IL-4-treated ESCs. Among them were VCAM1 and M-cadherin, as previously we found that these factors are not present in undifferentiated ESCs, which may limit their ability to fuse with myoblasts [18]. The analysis also encompassed integrin β1 (*Itgb1*) and integrin α4 (*Itga4*), as it was previously reported that IL-4 promotes expression of *Itgb1* in B cells and macrophages, while *Itga4* is necessary to form functional complexes with VCAM1 [20]. Since expression of cell membrane factors significantly changes during PSC differentiation [21,22,23] we performed the analysis using both undifferentiated ESCs as well as those differentiating in EBs for 2, 5, or 7 days (described as EB2, EB5, and EB7, respectively; experimental scheme in Supplementary Figure S1B). To precisely assess the influence of IL-4 on ESCs, they were cultured under defined conditions, i.e., in the medium containing serum replacement (SR). 

**Figure 3:**
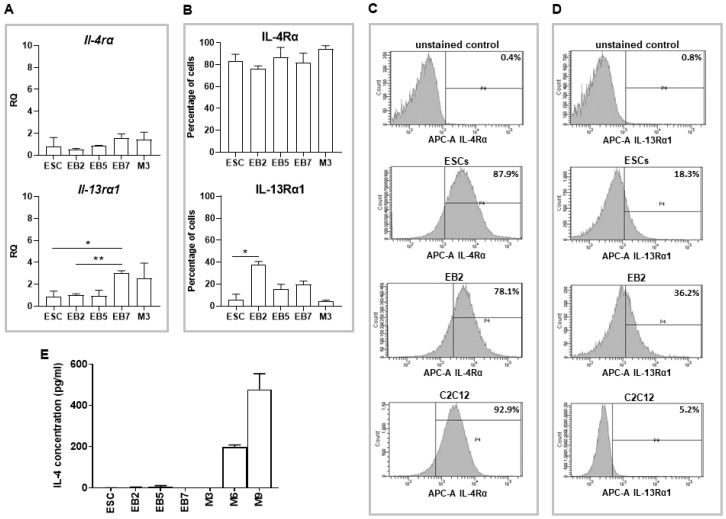
Analysis of expression of IL-4 and IL-4 type II receptor in ESCs differentiating in the EBs. (**A**) Expression of *Il-4rα* and *Il-13rα1* in undifferentiated ESCs, ESCs differentiating in EBs for 2 (EB2), 5 (EB5) and 7 (EB7) days, and proliferating C2C12 myoblasts at day 3 of the culture (M3). *β-actin* was used as a reference gene. RQ = 1 for the expression level detected in 13.5-day-old mouse embryo. Data are represented as the means of three independent experiments with standard deviations; * *p* < 0.05; ** *p* < 0.01. (**B**) Percentage of undifferentiated ESCs, cells obtained after disaggregation of EB2, EB5, and EB7 and proliferating C2C12 myoblasts synthesizing IL-4Rα or IL-13Rα1, analyzed with flow cytometry. * *p* < 0.05. (**C**,**D**) Representative histograms presenting percentage of cells synthesizing IL-4Rα (**C**) or IL-13Rα1 (**D**) in unstained ESCs (negative control, upper) and stained populations of either ESCs, EB2-derived cells, and C2C12 myoblasts. (**E**) IL-4 concentration in the medium collected from ESCs, ESCs differentiating in EBs, and C2C12 myoblasts. Data are represented as means of three independent experiments with standard deviations.

First, we verified whether ESCs differentiating in EBs synthesize endogenous IL-4 and express subunits of IL-4 type II receptor—i.e., are able to respond to IL-4 treatment. Results of qPCR analysis revealed that transcripts encoding *Il-4rα* and *Il-13rα1* subunits were present in all analyzed EBs at similar levels as detected in undifferentiated ESCs (Figure 3A). To assess the number of differentiating ESCs which could respond to IL-4 treatment, we disaggregated EBs and analyzed the presence of IL-4 type II receptor subunits in single cells. Flow cytometry analysis revealed that IL-4Rα was detected in the majority of undifferentiated and also differentiating ESCs, as well as in proliferating C2C12 myoblasts, which served as the positive control (Figure 3B–D). The percentage of cells in which IL-13Rα1 subunit was detected was much lower in comparison to IL-4Rα, but still substantial and increased during cell differentiation in EBs; approximately 40% of cells obtained from EB2 synthesized this receptor subunit (Figure 3B–D). Similarly to undifferentiated ESCs, cells differentiating in EBs did not synthesize endogenous IL-4, which was detected by ELISA in fusing C2C12 myoblasts and myotubes only (Figure 3E). Thus, these findings revealed that—similarly to undifferentiated ESCs—cells differentiating in EBs are also able to bind IL-4 but do not secrete this cytokine. 

**Figure 4:**
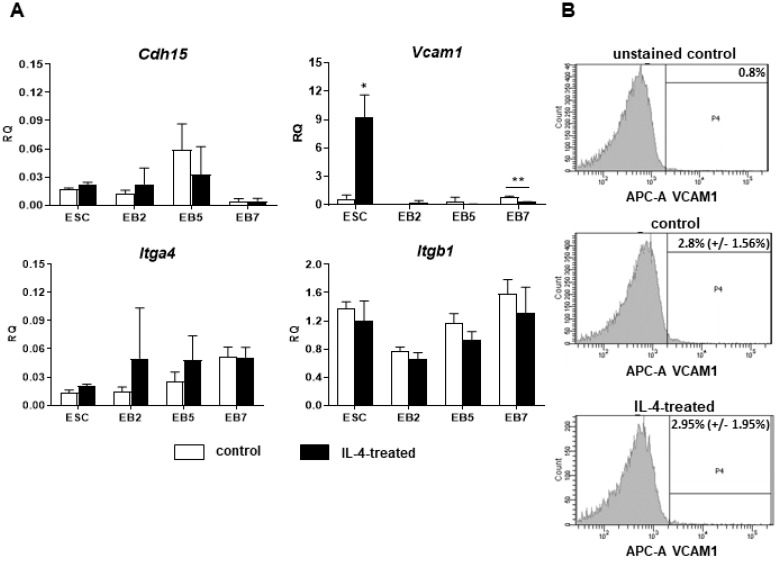
Analysis of IL-4 influence on expression of selected cell membrane proteins in ESCs. (**A**) Expression of *Cdh15*, *Vcam1*, *Itga4,* and *Itgb1* in undifferentiated and differentiating ESCs cultured with or without exogenous IL-4. *β-actin* was used as a reference gene. RQ = 1 for the expression level detected in 13.5-day-old mouse embryo. Data are presented as means of three independent experiments with standard deviations; * *p* < 0.05; ** *p* < 0.01. (**B**) Representative histograms presenting percentage of cells synthesizing VCAM1 in unstained control (upper), control (middle), or IL-4-treated (bottom) ESCs after 2 days of treatment, analyzed with flow cytometry.

Next, we focused on the expression of selected cell membrane factors, such as disintegrin and metalloproteinase domain-containing protein 9 (ADAM9), CD9, M-cadherin, VCAM1, integrin β1, integrin α4, and IL-4 type II receptor subunits in undifferentiated and differentiating ESCs cultured under defined conditions in the presence or absence of exogenous IL-4. ΔCt values obtained for 13.5-day-old mouse embryo, which served as a reference sample, indicated that *Il4rα*, *Itgb1*, *Itga4*, *Cd9*, *Cdh15* (encoding M-cadherin), and *Adam9* were expressed at moderate levels (ΔCt values range: 6–11) while expression of *Il13rα1* and *Vcam1* was low (ΔCt values over 11) in mouse embryo (data not shown). The expression level of all analyzed factors was low in ESCs and did not significantly change during their differentiation (Figure 4A). IL-4 significantly increased the expression level of *Vcam1* in undifferentiated ESCs only (Figure 4A); however, no changes in the number of cells synthesizing this protein were detected by flow cytometry analysis (Figure 4B). Altogether, our results indicated that increased frequency of fusion between ESCs and myoblasts was not caused by changes in the expression of cell membrane proteins such as *Adam9*, *Cd9*, *Cdh15*, *Vcam1*, *Itgβ1*, *Itgα4*, *Il4rα* and *Il13rα1*.

### 2.4. Influence of IL-4 on Mesodermal and Myogenic Regulator Expression in ESCs

We have analyzed the expression of only few selected cell membrane proteins and for this reason we cannot exclude possibility that IL-4 modifies expression of others. However, since IL-4 treatment had no significant effects on cell membrane protein expression, in the next step we focused on the potential influence of this cytokine on mesodermal/myogenic ESC differentiation. To address this issue, we assessed the expression level of mRNAs encoding Mesogenin1 (Msgn1) and Platelet-derived growth factor receptor α (PDGFRα)*,* the markers of presomitic and paraxial mesoderm, respectively, which are sources of skeletal muscle lineage. We also assessed the expression of *Flk1*, the marker of lateral mesoderm, a source of other lineages such as blood [24]. Next, we analyzed *Pax3* and *Pax7*, markers of skeletal muscle precursor cells formed during embryonic myogenesis, as well as *Myf-5* and *Myod*, markers of committed myogenic cells, acting downstream of *Pax3* and *Pax7* [25]. In addition, we analyzed the expression of *Nanog*, a key pluripotency marker, characteristic of undifferentiated PSCs [26]. The expression of all indicated markers was assessed in both ESCs and EBs cultured under defined conditions—i.e., in SR—either in the presence or the absence of exogenous IL-4 (experimental scheme in Supplementary Figure S1B). 

**Figure 5:**
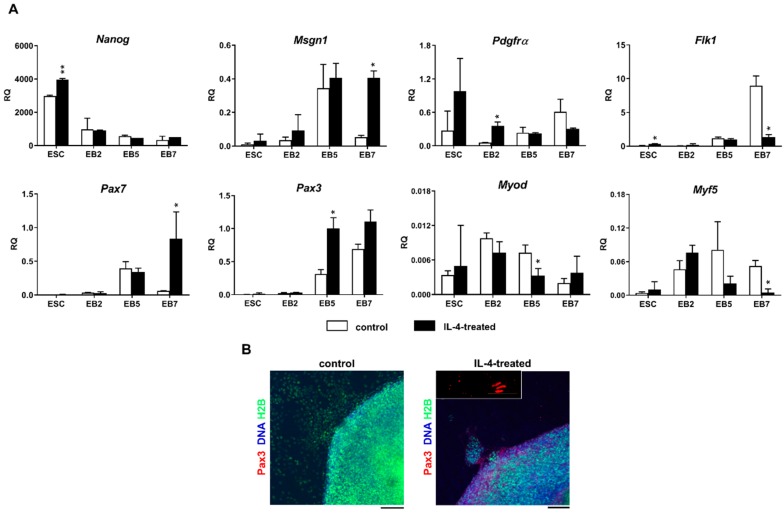
Analysis of IL-4 influence on selected mesodermal and myogenic marker expression in ESCs. (**A**) Expression of *Nanog*, *Msgn1*, *Flk1*, *Pdgfrα*, *Pax3*, *Pax7*, *Myod*, and *Myf5* in undifferentiated and differentiating ESCs cultured with (IL-4-treated) or without (control) exogenous IL-4. β-*actin* was used as a reference gene. RQ = 1 for the expression level detected in 13.5-day-old mouse embryo. Data are presented as means of three independent experiments with standard deviations; * *p* < 0.05; ** *p* < 0.01. (**B**) Representative images of immunocytochemistry analysis of Pax3 in IL-4-treated and control EB7. Scale bar: 100 μm.

Analysis of ΔCt values in the reference sample, 13.5-day-old mouse embryo indicated that expression of *Pdgfrα, Flk1, Pax3, Pax7, Myf-5,* and *Myod* was moderate while expression of *Nanog* and *Msgn1* was low (data not shown). The level of *Nanog* expression was high in undifferentiated ESCs and then declined during their differentiation, regardless of IL-4 treatment (Figure 5A). The expression of *Msgn1*, the presomitic mesoderm marker, was negligible in undifferentiated ESCs but significantly increased in IL-4-treated EBs, as compared to control EBs, and stayed high in both EB5 and EB7 (Figure 5A). *Pdgfrα* was expressed at low levels in both undifferentiated and differentiating ESCs, but IL-4 treatment led to the transient—observed in EB2 only—yet significant increase in its expression (Figure 5A). The expression of the lateral mesoderm marker, *Flk1*, was negligible in undifferentiated ESCs, EB2, and EB5, but significantly increased in EB7; however, only in cells cultured in the absence of exogenous IL-4. Thus, IL-4 decreased *Flk-1* expression in EB7 (Figure 5A). The initial expression level of all analyzed myogenic markers—i.e., *Pax3*, *Pax7*, *Myf-5*, and *Myod*—in undifferentiated ESCs was low but increased during their differentiation (Figure 5A). IL-4 treatment significantly elevated *Pax3* and *Pax7,* but decreased *Myf-5* and *Myod* expression in EB5 and EB7 (Figure 5A). Immunocytochemistry analysis revealed that Pax3, the most important marker of skeletal muscle precursor cells, was also present at the protein level in IL-4 treated EB7, however only in the subset of analyzed cells (Figure 5B). 

To verify that observed effects were indeed caused by IL-4 treatment we cultured EBs in the presence of IL-4 and antibodies targeting either IL-4 or IL-4Rα and found that the profile of analyzed gene expression was similar to control cells (Supplementary Figure S4). When cells were cultured in the presence of IL-4 and control anti-IgG antibody, the gene expression profile was similar to IL-4 treated cells (Supplementary Figure S4). Thus, IL4 action indeed results in increased level of expression of early myogenic genes such as Mesogenin1, Pax3, and Pax7 in differentiating embryonic stem cells.

## 3. Discussion

In our previous study, we showed that undifferentiated mouse ESCs fuse with skeletal muscle myoblasts, which leads to the appearance of MRFs, such as MyoD and myogenin, in ESC-derived nuclei localized in hybrid myotubes [18]. Fusion of ESCs with myoblasts was a prerequisite for their myogenic conversion as no significant effect was noticed for ESCs which did not participate in hybrid myotube formation. Thus, factors released by differentiating myoblasts and present in culture medium promoting myoblast differentiation were not sufficient to induce myogenic conversion of ESCs until other factors—e.g., pre-treatment with 5-azactidine, hemimethylating agent—were used. Currently available protocols enable efficient myogenic conversion of PSCs by modulating crucial pathways regulating myogenesis, such as those dependent on Wnt, FGF-2, or BMP-4 [4]. However, while development of functional skeletal muscle during mouse embryogenesis takes circa 10 days, abovementioned protocols usually require much longer—e.g., 5 times as much time—to generate myogenic cells from PSCs [5]. Moreover, in many protocols animal-derived reagents are used while generation of clinically-relevant human cells should be conducted under defined and animal-free conditions. For this reason, further improvement of protocols leading to myogenic conversion of PSCs is needed. To address this issue, we investigated IL-4 as a potential booster of myogenic conversion for mouse ESCs. 

As mentioned before, fusion between undifferentiated ESCs and myoblasts leading to formation of hybrid myotubes occurs with very low frequency (no higher than 1%; [18]). Since IL-4 promotes fusion of myoblasts with nascent myotubes, we hypothesized that co-culture of undifferentiated ESCs and myoblasts in the presence of this cytokine may enhance formation of hybrid myotubes. Initial experiments confirmed the presence of IL-4 type II receptor subunits in myoblasts, which is in line with the previous report [8] and revealed the presence of both subunits in undifferentiated as well as differentiating ESCs. We also showed that ESCs do not express IL-4, while secretion of this cytokine significantly increases during myoblast differentiation progression, together with the increase in IL-4Rα subunit expression. Our results partially stay in agreement with a report by Schmitt and co-workers who showed that IL-4 is not produced either in undifferentiated or differentiating ESCs, while the receptor for this cytokine appears in differentiating but not undifferentiated ESCs [27]. However, the type of IL-4 receptor analyzed in the indicated study was not specified. We found that γC, the subunit of IL-4 type I receptor, is not expressed in undifferentiated ESCs, so this receptor type is indeed not present in these cells in contrast to type II receptor.

The co-culture of undifferentiated ESCs and myoblasts in the presence of IL-4 resulted in a higher number of hybrid myotubes than in the control variant, but it should be noted that such myotubes were still scarce and made up to no more than 3% of all myotubes formed in the co-culture. We also noticed the increase in hybrid myotube number in the IL-4-treated co-culture of proliferating and differentiating myoblasts in comparison to the control, but due to high standard deviations observed differences were not statistically significant. IL-4 did not influence the co-culture of myoblasts with MEFs, which is in agreement with the fact that we did not detect IL-4 receptor in MEFs. We also showed that IL-4 has no impact on proliferation of any cell types used in the experiments—i.e., undifferentiated and differentiating ESCs, MEFs, and myoblasts—cultured separately as well as in the co-cultures with C2C12 myoblasts. To our knowledge, the lack of IL-4 influence on PSC and MEF proliferation has not been described yet, while Lafreniere and co-workers already reported that IL-4 does not impact human myoblast proliferation [17]. Altogether, these results indicate that in contrast to immune cells, such as T cells, B cells, or mast cells [9,10,11,28], IL-4 does not influence the proliferation of PSCs, MEFs or skeletal muscle myoblasts. Although this statement has not been verified by us, one can hypothesize that the influence of IL-4 on cell proliferation may depend on the type of IL-4 receptor present in the cell membrane. While immune cells express IL-4 type I receptors, both ESCs and myoblasts are characterized by the presence of IL-4 type II receptor. 

Since IL-4 had no significant effect on the fusion index in the co-culture of ESCs and myoblasts, but at the same time moderately elevated the number of hybrid myotubes formed by these cells, we concluded that this effect is mainly linked to the IL-4 impact on ESCs. To further address this issue, we cultured undifferentiated ESCs in the presence or absence of IL-4 under defined culture conditions—i.e., in the medium containing SR instead of serum. Moreover, we included ESCs differentiating in EBs in this analysis, as the effect of IL-4 action was noticed only after 9 days of the ESC-myoblast co-culture when first stages of ESC differentiation could have already occurred. Since several reports already described the influence of IL-4 on cell membrane proteins, e.g., [13,14,15,16,17], we performed analysis of selected genes encoding cell membrane proteins but did not observe any significant effects except for transient elevation of *Vcam1* expression in undifferentiated ESCs. Changes in gene expression were, however, not accompanied by the synthesis of VCAM1 protein in IL-4-treated ESCs. Surprisingly, we found that IL-4 treatment results in the significant increase in the expression level of mesodermal and early myogenic markers: *Msgn1*, *Pdgfra*, *Pax3*, and *Pax7* in differentiating ESCs. IL-4 effect was mainly visible in EB5 and EB7, which is in agreement with reports indicating appearance of the first PSC-derived myogenic cells in EBs cultured for 5 and 7 days [2]. Since IL-4 also decreased the levels of late myogenic regulators such as *Myf-5* and *MyoD* in differentiating ESCs, we speculated that this cytokine may enhance early stages of myogenic conversion of PSCs. Decrease in *Myf-5* and *MyoD* expression in IL-4-treated ESCs turned out to be transient as the expression of these genes increased in outgrowths derived from such EBs and was even higher than in control—i.e., untreated EBs (data not shown). 

Nevertheless, our results indicate that IL-4 affects competence of mouse ESCs for myogenic conversion by increasing their participation in hybrid myotube formation during co-culture with C2C12 myoblasts, as well as by elevating the expression level of genes encoding mesodermal and early myogenic markers—such as *Msgn1*, *Pax3*, and *Pax7*. Since the observed effect was rather modest, IL-4 treatment is not sufficient to induce robust PSC myogenic differentiation alone but it could be implemented to other protocols to further improve such conversion. To our knowledge, this effect has not been reported so far, while the increased frequency of fusion between myoblasts and stem cells co-cultured in the presence of IL-4 was already described for bone marrow mesenchymal stem cells (BM-MSCs) [13]. Interestingly, our recent research revealed that pre-treatment of BM-MSCs with IL-4 results in higher frequency of hybrid myotube formation in the subsequent co-culture of these cells with myoblasts but does not increase the expression of selected cell membrane factors engaged in adhesion and fusion (Kasprzycka et al., submitted). This may indicate that IL-4 may impact different types of stem cells by the same, yet unknown mechanism, enhancing their competence for fusion and myogenic conversion.

## 4. Materials and Methods

### 4.1. Animals

Animal care and all experimental procedures were approved by the Local Ethics Committee No. 1 in Warsaw, Poland, according to the European Union Directive on the approximation in laws, regulations, and administrative provisions of the Member States regarding protection of animals used for experimental and scientific purposes (permit number: 300/2012; 24 May 2012; Poland).

### 4.2. Cell Culture

Mouse embryonic fibroblasts (MEFs) were isolated from 13.5-day-old embryos obtained after mating C57BL6N mice. MEFs were cultured in Dulbecco’s modified Eagle’s medium (DMEM) with 4.5 mg/L glucose (Thermo Fisher Scientific, Waltham, MA, USA), supplemented with 10% heat inactivated fetal bovine serum (FBS, Thermo Fisher Scientific) as well as 50 U/mL penicillin (Thermo Fisher Scientific) and 50 µg/mL streptomycin (Thermo Fisher Scientific; hereafter referred to as antibiotics). After reaching confluence MEFs were inactivated with mitomycin C (0.01 mg/mL in MEF culture medium; Sigma-Aldrich) and next used as a feeder layer for ESC culture. Mouse ESCs constitutively expressing histone H2B fused with GFP (hereafter referred to as ESCs-GFP) were provided by Dr. Kat Hadjantonakis, Memorial Sloan Kettering Cancer Center New York [29]. 

Inactivated MEFs were plated onto culture dishes coated with 1% gelatin (Sigma-Aldrich) in DMEM with 4.5 mg/L glucose (Thermo Fisher Scientific), supplemented with 10% heat inactivated FBS, and antibiotics. Next, GFP-expressing ESCs were seeded onto a MEF layer and cultured in medium consisting of Knockout DMEM (Thermo Fisher Scientific) supplemented with 15% ESC-qualified FBS (Thermo Fisher Scientific), 0.1 mM nonessential amino acids (Thermo Fisher Scientific), 2 mM L-glutamine (Thermo Fisher Scientific), 0.1 mM β-mercaptoethanol (Sigma-Aldrich, St. Louis, MO, USA), antibiotics, and 500 U/mL leukemia inhibitory factor (LIF; Chemicon) to maintain them in undifferentiated pluripotent state.

C2C12 myoblasts (European Collection of Cell Cultures ECACC no. 91031101, passage no. 13) were cultured in medium supporting their proliferation, i.e., high-glucose DMEM supplemented with 10% FBS and antibiotics. After 3 days of culture, this medium was switched to the one promoting myoblast differentiation and fusion—i.e., DMEM supplemented with 5% horse serum (HS, Thermo Fisher Scientific), and antibiotics [30]. 

Primary mouse myoblasts were obtained as a result of differentiation of satellite cells isolated from hind limb muscles: flexor digitorum brevis, extensor digitorum longus, tibialis anterior, and Soleus, as described previously [31]. Briefly, muscles were dissected from the hind limb of 3-month-old C57BL6N males. Isolated muscles were digested with 0.2% type I collagenase (Sigma Aldrich) in low-glucose DMEM at 37 °C for 90 min. Single myofibers were obtained by gentle trituration of muscle after digestion. Next, satellite cells were liberated from myofibers with a 22 G needle and plated onto dishes coated with Matrigel (BD Biosciences), diluted 1:10 in low-glucose DMEM. Satellite cell derived primary myoblasts were cultured in low-glucose DMEM supplemented with 10% HS, 20% FBS, 0.5% Chicken Embryo Extract (SeraLab, Haywards Heath, West Sussex, UK), and antibiotics on matrigel-coated dishes under standard conditions (37 °C, 5% CO_2_).

### 4.3. In Vitro Differentiation of ESCs

GFP-expressing ESCs were differentiated in embryoid bodies (EBs). EBs were generated using the hanging drops technique. ESCs were separated from MEFs by the pre-plating procedure. Briefly, ESC and MEF suspension was plated on gelatin-coated dishes and incubated at 37 °C for 20 min. After the incubation, unattached ESCs were transferred to a new culture plate. The procedure was repeated thrice. Next, 30 μL drops of medium for ESC culture, described above but devoid of LIF and supplemented with 15% Serum Replacement (SR, Thermo Fisher Scientific) instead of FBS and containing 800 ESCs were placed onto the bottom of covers of culture dishes filled with phosphate-buffered saline (PBS). At day 2 of culture in hanging drops, EBs were transferred to low-adhesive culture dishes (Medlab, Raszyn, Poland), allowing their culture in suspension for further 3 or 5 days. After 2, 5, of 7 days of differentiation, EBs, referred to as EB2, EB5, or EB7, respectively, were collected for further analysis.

### 4.4. ESC and C2C12 Myoblast Co-Culture

Before co-culture experiments, GFP-expressing ESCs were separated from MEFs by the preplating method. Next, 15 × 10^3^ ESCs were collected and seeded onto 15 × 10^3^ fusing C2C12 myoblasts (at day 4 of C2C12 myoblast culture). Differentiating C2C12 myoblasts cultured with 15 × 10^3^ proliferating C2C12 myoblasts or with 15 × 10^3^ MEFs served as a positive or negative control, respectively. In control variants, proliferating C2C12 myoblasts or MEFs were labeled with the Cell TrackerTM Orange CMRA reagent according to the manufacturer’s protocol (Thermo Fisher Scientific) prior to seeding on the layer of differentiating C2C12 myoblasts. Co-cultures were conducted in high-glucose DMEM supplemented with 5% HS and antibiotics. Cells were analyzed at day 3, 6, or 9. Cells fixed with cold methanol were stained with Giemsa and May-Grunwald dyes, while cells fixed with 3% PFA were subjected to immunostaining analysis. 

### 4.5. IL-4 Treatment

All co-culture variants (C2C12-ESC, C2C12-C2C12, and C2C12-MEF) were cultured in high-glucose DMEM supplemented with 5% HS, antibiotics, and mouse IL-4 recombinant protein (Sigma-Aldrich) at final concentration of 5 ng/mL. Additionally, C2C12-ESC co-cultures were cultured in the presence of 5 ng/mL IL-4 and either 5 µg/mL or 10 µg/mL of anti-IL-4 antibody (I7034 Sigma Aldrich), or 5 ng/mL or 10 ng/mL of anti-IL-4Rα antibody (NBP1-00884 Novus Biologicals; Centennial, CO, USA). In control variants, cells were co-cultured in the presence of IL-4 and control antibody (donkey anti-rabbit IgG Thermo Fisher Scientific) or in the presence of antibodies only. Cells were collected and analyzed at day 3, 6, or 9 of IL-4 treatment. In all experiments, cells cultured without exogenous IL-4 served as control. 

For the experiments on IL-4 influence on in vitro ESC differentiation, ESCs and EBs were cultured in the medium supplemented with 15% SR, as described above, with exogenous IL-4 at the final concentration of 5 ng/mL. Medium was changed at day 2 and 5. Additionally, ESCs and EBs were cultured in the presence of 5 ng/mL IL-4 and either 5 µg/mL or 10 µg/mL of anti-IL-4 antibody (I7034 Sigma Aldrich), or 5 ng/mL or 10 ng/mL of anti-IL-4Rα antibody (NBP1-00884 Novus Biologicals). In control variants, cells were co-cultured in the presence of IL-4 and control antibody (donkey anti-rabbit IgG Thermo Fisher Scientific) or in the presence of antibodies only. EBs were collected and analyzed at day 2, 5, or 7. ESCs were collected and analyzed at day 2 of treatment. In all experiments, cells cultured without exogenous IL-4 served as control. 

### 4.6. RNA Isolation and qPCR Analysis

Total RNA was isolated from undifferentiated GFP-expressing ESCs, EB2, EB5, EB7, C2C12, and primary myoblasts at four stages of their culture as well as from 13.5-day-old mouse embryos using a High Pure RNA Isolation Kit (Roche) and transcribed to cDNA with RevertAid First Strand cDNA Synthesis Kit (Thermo Fisher Scientific) according to the manufacturer’s protocols. qPCR analysis was performed using specific TaqMan^®^ probes (Thermo Fischer Scientific): Mm012575139 (*Il4rα*), Mm00446726 (*Il13rα1*), Mm02019550_s1 (*Nanog*), Mm01222421 (*Flk1*), Mm00440701 (*Pdgfrα*), Mm00490407_s1 (*Msgn1*), Mm00435493_m1 (*Pax3*), Mm01354484 (*Pax7*), Mm0435125 (*Myf-5*), Mm00440387_m1 (*Myod*), Mm00483191_m1 (*Cdh15*), Mm01320970_m1 (*Vcam1*), Mm01253230_m1 (*Itgb1*), Mm01277951_m1 (*Itga4*), Mm00475770_m1 (*Adam9*), Mm00514275_g1 (*Cd9*), Mm01205647_g1 (*Actb*), the TaqMan Gene Expression Master Mix (Thermo Fischer Scientific) and LightCycler 96 instrument (Roche). Data were collected and analyzed with LightCycler 96 SW1.1 software (Roche). For each analysis, three independent experiments were performed. ΔΔCt analysis was performed according to Livak and Schmittgen [32].

### 4.7. Index of Fusion

Control or IL-4-treated co-cultures of differentiating C2C12 myoblasts and either ESCs or MEFs or proliferating C2C12 myoblasts were fixed in cold methanol for 10 min at 4 °C at day 3, 6, or 9 of the co-culture. Next, cells were stained with Giemsa and May–Grunwald dyes according to the manufacturer’s protocol (Merck, Darmstadt, Germany) and examined with 20× objectives on Eclipse TE200 microscope (Nikon) in order to calculate the index of fusion. The index of fusion is the ratio of nuclei localized in multinucleated myotubes compared to the number of all observed nuclei. Images were taken with DXM 1200 digital camera and analyzed using NIS Elements F 2.30 software (Nikon, Tokyo, Japan). Ten representative fields of view of control and IL-4-treated co-cultures at each time point were analyzed. 

### 4.8. Immunolocalization

To visualize IL-4, undifferentiated ESCs and myoblasts were incubated with Brefeldin A (Sigma-Aldrich) at concentration of 10 µg/mL prior to the fixation, at 37 °C for 3 h and fixed with 3% PFA at room temperature for 10 min. This procedure enables studying secreted proteins within fixed cells. Subsequently, all analyzed cell types fixed with 3% PFA were permeabilized with 0.1% Triton-X 100 (Sigma-Aldrich) at room temperature for 5 min. Nonspecific antibody binding was blocked by incubation in 3% bovine serum albumin (BSA, Sigma-Aldrich) in PBS for 1 h at room temperature. Next, primary antibodies were diluted in 0.5% BSA in PBS and incubated with cells at 4 °C overnight. Primary antibodies against the following antigens were used: IL-4Rα (diluted 1:50, Novus Biologicals NBP1-00884), IL-13Rα1 (diluted 1:50, Novus Biologicals NBP1-61690), IL-4 (diluted 1:50, Novus Biologicals NB100-64798), Pax3 (diluted 1:50, Developmental Studies Hybridoma Bank; Iowa City, IA, USA), skeletal muscle marker (12/101; diluted 1:50, Developmental Studies Hybridoma Bank). Afterwards, specimens were incubated with appropriate secondary antibodies conjugated with Alexa Fluor 594 (Thermo Fisher Scientific) diluted 1:200 in 0.5% BSA in PBS for 1.5 h, and DRAQ5 diluted 1:1000 in PBS at room temperature for 10 min. Specimens were washed twice with PBS and mounted using Fluorescent Mounting Medium (Dako, Glostrup, Denmark). The specimens were analyzed using Axiovert 100 M scanning confocal microscope (Zeiss, Jena, Germany) with LSM 510 software. 

### 4.9. FACS Analysis

For FACS analysis, ESCs were trypsynized and purified from MEFs using the preplating method and EBs were disaggregated with Enzyme-Free Hanks’-based Cell Dissociation Buffer (Thermo Fisher Scientific). The obtained single cell suspension was incubated with 0.1% BSA and 1% FBS in PBS at room temperature for 30 min. Next, cells were incubated with primary antibodies on ice for 30 min. Antibodies against following antigens were used: IL-4Rα (diluted 1:50, Novus Biologicals NBP1-00884, Centennial, CO, USA), IL-13Rα1 (diluted 1:50, Novus Biologicals NBP1-61690), and VCAM1 (diluted 1:100, R&D Systems MAB6432, Minneapolis, MN, USA). Cells were washed with PBS once. Afterwards, cells were incubated with appropriate secondary antibody conjugated with APC-A (diluted 1:100, Thermo Fisher Scientific) at 4 °C for 30 min. Cells were fixed with 0.1% PFA at 4 °C for 10 min and analyzed with LSRFortessa cytometer (Becton Dickinson, Warsaw, Poland) and Diva 6.1 software.

### 4.10. IL-4 Concentration

IL-4 concentration in collected culture supernatants was studied by ELISA analysis with Quantikine^®^ ELISA (R&D Systems, M4000B) according to the manufacturer’s protocol. The plate was analyzed with Gen5 Microplate Reader and Image software. 

### 4.11. Statistical Analysis

All experiments were performed at least three times. Data are presented as mean ± standard deviation (SD) from three independent experiments, and Student’s *t*-test was used for statistical analysis, *p* < 0.05 was considered statistically significant and marked with asterisks. 

## Supplementary Materials

The supplementary materials are available online at https://www.mdpi.com/1422-0067/20/16/3932/s1.

## Abbreviations

ADAM9	a disintegrin and metalloproteinase domain-containing protein 9
BM-MSCs	bone marrow derived mesenchymal stem cells
BSA	bovine serum albumin
DMD	Duchenne muscular dystrophy
DMEM	Dulbecco’s modified Eagle’s medium
EBs	embryoid bodies
ESCs	embryonic stem cells
ESCs-GFP	embryonic stem cells expressing green fluorescent protein
FBS	fetal bovine serum
FGF-2	fibroblast growth factor 2
HS	horse serum
IGF-1	insulin-like growth factor 1
IL-4	interleukin 4
iPSCs	induced pluripotent stem cells
LIF	leukemia inhibitory factor
MEFs	mouse embryonic fibroblasts
MHC	myosin heavy chain
MRFs	muscle regulatory factors
PBS	phosphate buffer saline
PDGFRα	platelet derived growth factor receptor α
PSCs	pluripotent stem cells
SR	serum replacement
SRF	serum response factor
VCAM1	vascular cell adhesion molecule 1
