# Supplementary material for: Interleukin 4 Moderately Affects Competence of Pluripotent Stem Cells for Myogenic Conversion
Source: Int J Mol Sci. 2019 Aug 13;20(16):3932. doi: 10.3390/ijms20163932 (PMC6720909; doi:10.3390/ijms20163932)
Supplement: Supplementary file 1 [file ijms-20-03932-s001.pdf]

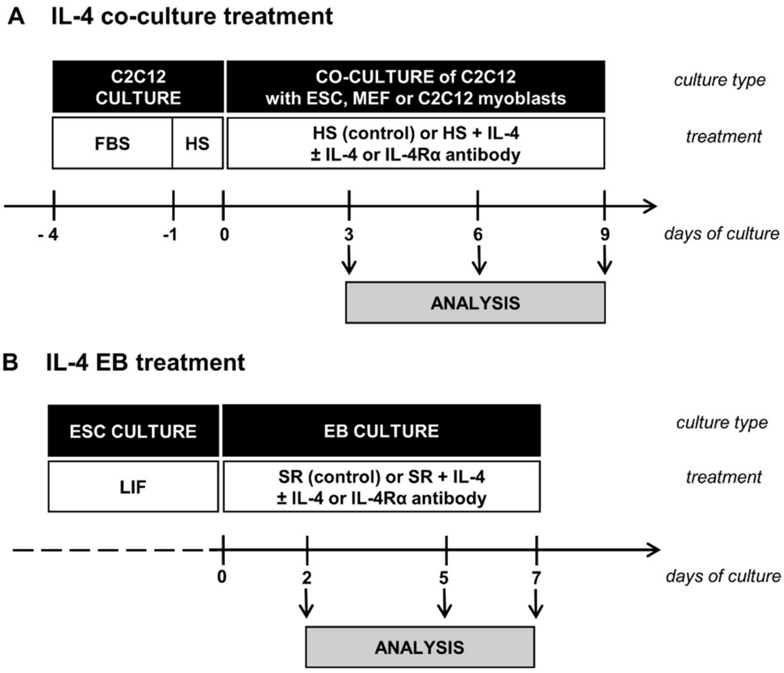

Supplementary Figure S1. Experimental schemes.

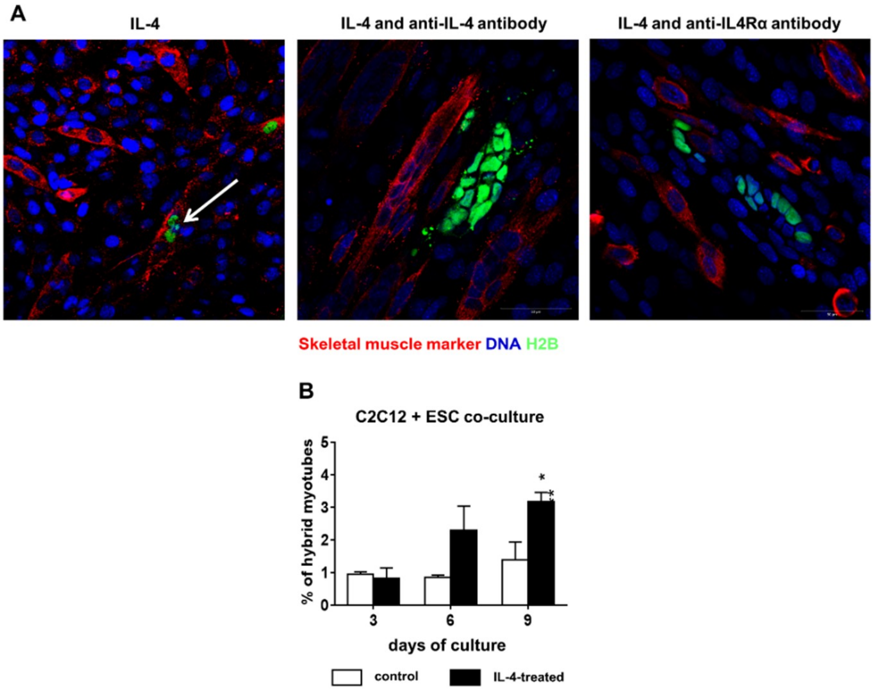

Supplementary Figure S2. Hybrid myotube formation in co-culture of ESCs and C2C12 myoblasts. (A) Representative images of immunocytochemistry analysis of ESCs and C2C12 myoblasts co-cultured in the indicated conditions and analyzed after 9 days. Myotubes were identified on the basis of skeletal muscle marker immunolocalization. Hybrid myotubes were identified on the basis of GFP expression in myotube nuclei (white arrow indicate hybrid myotube). (B) Frequency of hybrid myotube contribution to total number of myotubes in the co-culture of ESCs and C2C12 myoblasts. Data are presented as the means of three independent experiments with standard deviations; $*p < 0.05$.

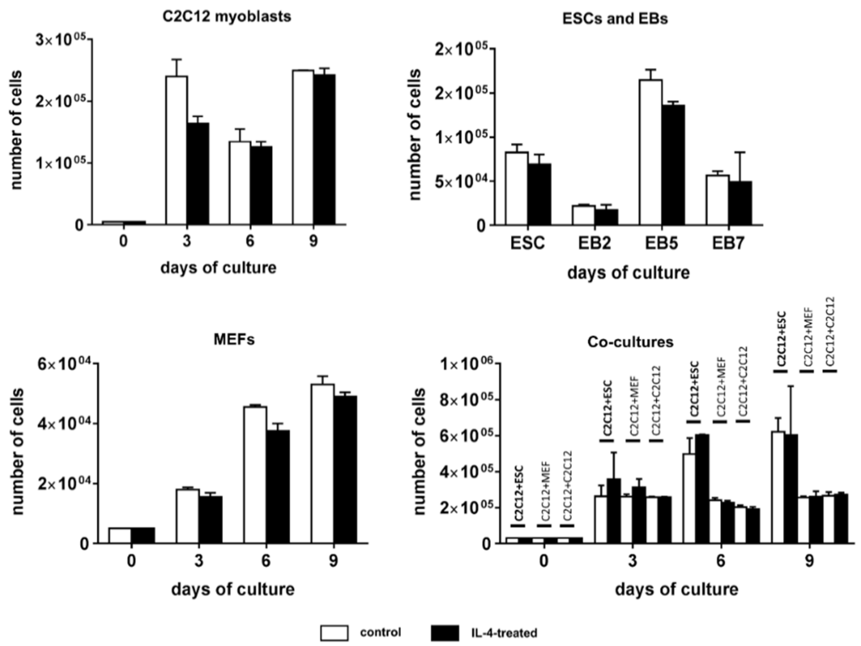

Supplementary Figure S3. **Influence of IL-4 on cell proliferation.** Graphs present number of cells cultured in the presence or absence of exogenous IL-4. Cell number was calculated at day 3, 6 and 9 of the culture for C2C12 myoblasts, MEFs, and all types of the co-culture, and at day 2, 5 and 7 for EBs. Data are shown as the mean of three independent experiments with standard deviations.

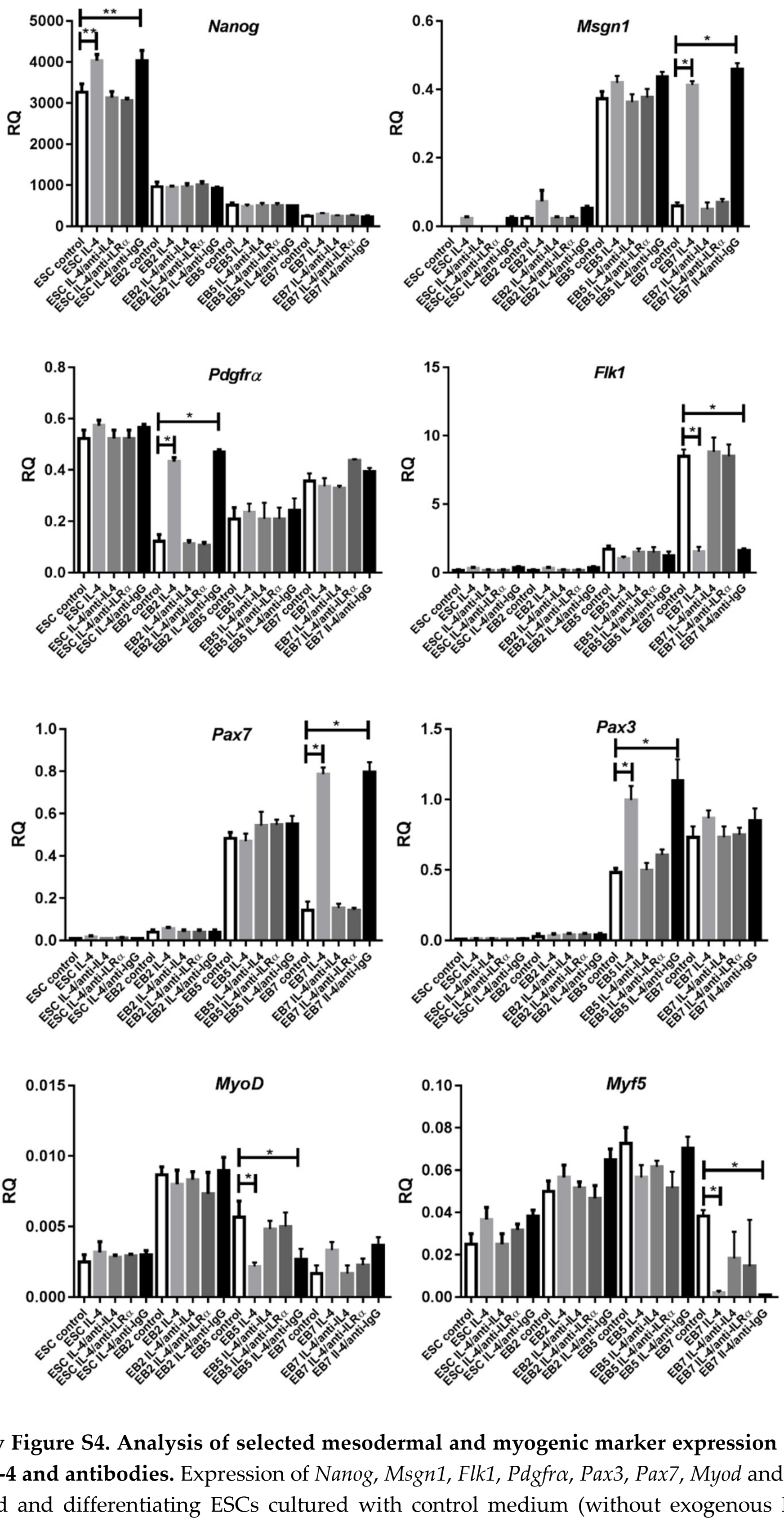

Supplementary Figure S4. Analysis of selected mesodermal and myogenic marker expression in ESCs treated with IL-4 and antibodies. Expression of *Nanog*, *Msgn1*, *Flk1*, *Pdgfrα*, *Pax3*, *Pax7*, *Myod* and *Myf5* in undifferentiated and differentiating ESCs cultured with control medium (without exogenous IL-4 nor antibodies) or in medium supplemented with IL-4 or in presence of IL-4 and either anti-IL4 or anti-IL4Rα or anti-IgG (control) antibody. β-*actin* was used as a reference gene. RQ=1 for the expression level detected in 13.5-day-old mouse embryo. Data are presented as means of two independent experiments with standard deviations; *$p < 0.05$; **$p < 0.01$.
